# Serum DJ-1 Is a Biomarker of Colorectal Cancer and DJ-1 Activates Mitophagy to Promote Colorectal Cancer Progression

**DOI:** 10.3390/cancers13164151

**Published:** 2021-08-18

**Authors:** William Tzu-Liang Chen, Han-Bin Yang, Tao-Wei Ke, Wen-Ling Liao, Shih-Ya Hung

**Affiliations:** 1 School of Medicine, China Medical University, Taichung 40402, Taiwan; wtchen@mail.cmuh.org.tw; 2 Department of Colorectal Surgery, China Medical University Hsinchu Hospital, Zhubei City 30272, Taiwan; 3 Ph.D. Program for Aging, China Medical University, Taichung 40402, Taiwan; alex23206567@yahoo.com.tw; 4 Division of Colorectal Surgery, China Medical University Hospital, Taichung 40447, Taiwan; D18047@mail.cmuh.org.tw; 5 Graduate Institute of Integrated Medicine, China Medical University, Taichung 40202, Taiwan; wl0129@mail.cmu.edu.tw; 6 Center for Personalized Medicine, Department of Medical Research, China Medical University Hospital, Taichung 40447, Taiwan; 7 Division of Surgery, Department of Medical Research, China Medical University Hospital, Taichung 40447, Taiwan; 8 Graduate Institute of Acupuncture Science, China Medical University, Taichung 40402, Taiwan

**Keywords:** autophagy, biomarker, colorectal cancer, DJ-1, mitophagy

## Abstract

**Simple Summary:**

Colorectal cancer is common cancer, and currently used serum markers for detecting colorectal cancer lack excellent diagnostic accuracy. In the present study, we collected matched tumor and adjacent normal tissues and serum from patients and cancer cells to demonstrate the clinical value of DJ-1 in colorectal cancer and the role of DJ-1-induced mitophagy in colorectal cancer progression. Our data indicate that DJ-1 might be clinically valuable as serum and tissue biomarkers for predicting the TNM (tumor-node-metastasis) stage in colorectal cancer patients. Besides, DJ-1 knockdown enhanced intracellular reactive oxygen species generation and damaged mitochondrial accumulation and mitophagy inhibition in metastatic colorectal adenocarcinoma cells. Since DJ-1-induced mitophagy promotes tumor progression, DJ-1 inhibition is a potential therapeutic strategy for colorectal cancer treatment.

**Abstract:**

Colorectal cancer is the second most common cancer and the third cancer-associated death in Taiwan. Currently used serum markers for detecting colorectal cancer lack excellent diagnostic accuracy, which results in colorectal cancer being often recognized too late for successful therapy. Mitophagy is the selective autophagic degradation of damaged or excessive mitochondria. DJ-1 is an antioxidant protein that attenuates oxidative stress and maintains mitochondrial quality through activating mitophagy. Mitophagy activation contributes to anti-cancer drug resistance. However, the role of DJ-1-induced mitophagy in colorectal cancer progression remains unclear. In the present study, we collected matched tumor and adjacent normal tissues and serum from patients and cancer cells to demonstrate the clinical value and physiological function of DJ-1 in colorectal cancer. We found that DJ-1 increased in tumor tissues and serum; it was positively correlated with TNM (tumor-node-metastasis) stages of colorectal cancer patients. Through stable knockdown DJ-1 expression in metastatic colorectal adenocarcinoma cells SW620, DJ-1 knockdown inhibited cancer cell survival, migration, and colony formation. In SW620 cells, DJ-1 knockdown induced an incomplete autophagic response that did not affect ATP production; DJ-1 knockdown enhanced intracellular reactive oxygen species generation and damaged mitochondrial accumulation and mitophagy inhibition. It suggests that DJ-1 knockdown inhibits mitophagy that causes metastatic colorectal adenocarcinoma cells to be unable to remove damaged mitochondria and further enhance cancer cell apoptosis. Our data indicate that DJ-1 might be clinically valuable as serum and tissue biomarkers for predicting the TNM stage in colorectal cancer patients. Since DJ-1-induced mitophagy promotes tumor progression, DJ-1 inhibition is a potential therapeutic strategy for colorectal cancer treatment.

## 1. Introduction

In 2020, colorectal cancer was the third most common cancer and cancer-related death in the United States [1]. In 2018, the cancer registry report of Taiwan shows that colorectal cancer was the second most common cancer and the third cancer-associated death in Taiwan [2]. The current TNM staging system for colorectal cancer is based on three elements: the penetration of the tumor into the intestinal wall (T), the number of positive lymph nodes present (N), and the presence of metastasis (M) [3]. About 22% of colorectal cancer patients are metastatic at initial diagnosis, and the patients with metastatic colorectal cancer face a poor prognosis and have a relative 5-year survival rate of 14% [4]. A study shows that the five-year recurrence rates in 871 TNM stage II and 265 TNM stage III colon cancer patients were 10% and 30%, respectively [3]. Additionally, the median overall survival of patients with metastatic colorectal cancer was about 30 months in recent clinical trials of first-line therapies [5]. Colorectal cancer progression is complex, including cancer cell proliferation, invasion, detachment, migration, adhesion, etc. Thus, early detection of colorectal cancer and elucidating detailed mechanisms underlying cancer progression are required to provide more reliable molecular targets for therapy and improve colorectal cancer prognoses.

Autophagy is a lysosomal degradative process that degrades and recycles the selected cytoplasmic substrates (e.g., obsolete cellular constituents, damaged organelles, abnormal protein aggregates, and viruses) by wrapping them into autophagosomes and fusing with lysosomes for substrate digestion by lysosomal hydrolases [6]. The process of autophagy includes autophagy induction, substrate recognition and selection, autophagosome biogenesis (phagophore nucleation/induction, phagophore elongation, substrate binding, and vacuole formation), autophagosome-lysosome fusion, and substrate degradation and recycling [6,7]. Over 30 genes participate in autophagy induction and autophagosome biogenesis [8]. Beclin 1 is a key element to regulate autophagy induction by interacting with several cofactors, including Vps34 (PI3KC3), Vps15, and Ambra1, to form the Beclin 1-Vps34-Vps15 core complex [9]. During autophagosome biogenesis, Atg5, Atg7, Atg16L, Atg10, and Atg12 regulate phagophore formation; LC3, Atg3, and Atg4B regulate vacuole formation [6,8]. However, the genes that regulate substrate binding during autophagosome biogenesis are still unclear. Sato et al. (2007) showed that autophagy is activated in colorectal cancers in vitro and in vivo, which may contribute to cancer cell survival in their microenvironment [10]. The PI3K/Akt/mTOR (mammalian target of rapamycin) signaling pathway is a well-known autophagy regulation pathway. Inhibition of the PI3K/Akt/mTOR signaling pathway activates autophagy in HT-29 human colorectal cancer cells [11].

Mitochondria are double membrane-bound organelles in the cytoplasm of cells, which participate in multiple cellular processes, including ATP production, calcium homeostasis, metabolic synthesis, apoptosis, reactive oxygen species (ROS) generation, etc. [12]. Mitophagy (mitochondria-specific autophagy) is the fundamental process of clearing damaged or excessive mitochondria by autophagy [13]. Damaged or dysfunctional mitochondria could harm the cell by generating excessive reactive oxygen species and releasing pro-apoptotic signals such as cytochrome c; eliminating the damaged or dysfunctional mitochondria by mitophagy prevents cell apoptosis [14]. Moderate metabolic and oxidative stress levels can stimulate mitophagy by inhibiting the mTOR pathway [15]. Defects in the mitophagy machinery have been proposed to contribute to breast cancer progression [13]. However, most colorectal cancer studies focus on anti-cancer drug-induced mitophagy, which is associated with anti-cancer drug resistance. The role of mitophagy in colorectal cancer progression remains unclear.

In neurons, DJ-1 (Parkinson’s disease-associated protein 7, PARK7; 20-kDa) is a neuroprotective protein that acts as an antioxidant protein by attenuating oxidative stress and maintaining mitochondrial quality through activating mitophagy [16,17]. DJ-1 overexpression induces ERK-dependent mitophagy against rotenone-induced neuronal apoptosis [18]. Loss of DJ-1 leads to loss of mitochondrial polarization, fragmentation of mitochondria, and accumulation of markers of autophagy (LC3 puncta and lipidation) around mitochondria in human dopaminergic cells [19]. Parkinson’s disease is described as a mitochondrial disease of aging, and DJ-1 mutation is associated with the disease pathogenesis in early-onset familial Parkinson’s disease [20]. Therefore, mitophagy activating by DJ-1 plays a neuroprotective role in neurodegenerative disorders such as Parkinson’s disease. In addition, Fun et al. (2008) show that DJ-1 overexpression decreases Bax expression and inhibits caspase activation, whereas DJ-1 knockdown increases Bax protein levels and accelerates caspase-3 activation and cell death after UV exposure [21]. DJ-1 is overexpressed in many malignant tumors, including ovarian carcinoma, breast cancer, leukemia, prostate cancer, cervical cancer, pancreatic cancer, and colorectal cancer [22,23,24,25,26,27]. As yet, the role of DJ-1-induced mitophagy in colorectal cancer carcinogenesis is unknown.

Oncogenes, tumor suppressors, and signaling pathways have recently been reported in colorectal cancer. However, DJ-1-induced mitophagy in colorectal cancer carcinogenesis remains poorly understood. Zhou et al. (2018) showed that DJ-1 promotes colorectal cancer progression by activating the PLAGL2/Wnt/BMP4 axis [26]. Zheng et al. (2018) demonstrated that DJ-1 activates the PI3K-Akt pathway to promote colon cancer survival [27]. Lin et al. (2018) showed that high expression of DJ-1 promotes growth and invasion via the PTEN-Akt pathway and predicts a poor prognosis in colorectal cancer [24]. Wang et al. (2020) showed that DJ-1 activates the PI3K/Akt/mTOR signaling pathways to promote colorectal cancer cell growth and metastasis in vitro and in vivo [28]. It is worth noting that the PI3K/AKT/mTOR signaling pathway activation is a well-known autophagy inhibition pathway; mitophagy can be stimulated by mTOR pathway inhibition [15,29]. In addition, DJ-1 overexpression activates mitophagy in dopaminergic neurons [18]. Interestingly, we found that DJ-1 has dual functions in activating mitophagy and promoting colorectal cancer carcinogenesis. As yet, no report study DJ-1-induced mitophagy in colorectal cancer progression. We aimed to determine whether the regulatory mechanism of DJ-1 in colorectal cancer progression is associated with mitophagy. A high concentration of DJ-1 is detected in the serum of pancreatic cancer, melanoma, and Parkinson’s disease [30,31,32,33]. However, whether serum DJ-1 level is correlated with the TNM stage of colorectal cancer has not been addressed. We first analyze the association between DJ-1 expressions and TNM stages in serum and tissue pairs of colorectal tumor tissues and adjacent normal tissues from patients to determine whether DJ-1 could serve as a serum biomarker in colorectal cancer. In addition, we elucidate the role of DJ-1 in tumor cell growth and migration, autophagosome biogenesis, and mitochondrial functions by knockdown DJ-1 in metastatic colorectal adenocarcinoma cells. We hope we can elucidate the clinical value and detailed molecular mechanisms about DJ-1 in colorectal cancer progression.

## 2. Materials and Methods

### 2.1. Microarray Analysis and Correlation Coefficients in Gene Expressions

The GDS2947 microarray gene expression dataset was downloaded from the National Center for Biotechnology Information (NCBI) Gene Expression Omnibus (https://www.ncbi.nlm.nih.gov/sites/GDSbrowser?acc=GDS2947 accessed on 8 June 2021), and *DJ-1* mRNA expressions in 32 pairs of normal mucosa (≥2 cm from the site of the polyp) and pedunculated colorectal polyps (colorectal adenomas) of the same colon segment from 28 patients of the Belcolle City Hospital (Viterbo, Italy) were analyzed [34]. GEPIA (Gene Expression Profiling Interactive Analysis) was used for gene expression correlation analysis in tumor tissues from subjects with colon adenocarcinoma (http://gepia.cancer-pku.cn/ accessed on 8 June 2021). The Pearson correlation coefficients were used for analyzing the correlation of two gene expressions.

### 2.2. Serum and Paired Colorectal Tissues of Tumor Tissue (T) and Adjacent Normal Tissue (N) from Colorectal Cancer Patients

This study was approved by the Institutional Review Board of the China Medical University Hospital (CMUH104-REC3-096). Ninety-seven Taiwanese patients diagnosed with colorectal cancer and underwent surgery at China Medical University Hospital (Taichung, Taiwan) were enrolled in this study from 26 May 2016 to 2 December 2016. The characters of colorectal cancer patients can be found in Table S1. The serum and paired colorectal tissues of tumor tissue (T) and adjacent normal tissue (N) obtained from the same individual were stored at −80 °C before analysis. The medical records of each patient were reviewed by the medical doctor of the division of colorectal surgery, and pathologists classified the tumor stage of each patient according to the 7^th^ edition of the TNM Staging Manual of the American Joint Committee on Cancer [35].

### 2.3. RNA Extraction, Reverse-Transcription, and Real-Time Quantitative PCR

Total RNAs were extracted from each colorectal tissue pair for single-strand cDNA synthesis by reverse-transcription as described previously [36]. Real-time quantitative PCR was performed to analyze *DJ-1* and *β-actin* mRNA expressions by the StepOne Plus real-time PCR system (Applied Biosystems). The primer and probe sequences of *DJ-1* and *β-actin* for real-time quantitative PCR reactions are shown in Table 1. Thermal cycling conditions of real-time quantitative PCR and data calculation for *DJ-1* expression were described previously [36]. *DJ-1* expression was normalized with *β-actin* and then expressed as adjacent normal tissue relative to tumor tissue in each tissue pair by the ΔΔCt method described previously [36]. The relative expression level of *DJ-1* mRNA in each tissue pair of adjacent normal tissue (N) and colorectal tumor tissue (T) from TNM stage I, II, III, IV colorectal cancer patients can be found in Figure S1.

### 2.4. Western Blot and Immunohistochemistry (IHC) Staining

Western blot was used to analyze protein expressions in tissue pairs and cells. Tissues or cells were homogenized in radioimmunoprecipitation assay buffer containing 1% protease and phosphatase inhibitor cocktail (Hycell, Taipei, Taiwan). The protein concentration was determined by Pierce^TM^ BCA Protein Assay Kit (ThermoFisher Scientific, Waltham, MA, USA). SDS-PAGE separated protein at 30–50 μg under reducing conditions and electrotransferred onto Immobilon-PSQ^®^ PVDF membrane (Merck Millipore, Burlington, MA, USA). After being blocked with 5% nonfat milk in TBS-T (0.5% Tween 20 in 20 mM Tris and 137 mM NaCl) for 1 h at room temperature, the membranes were incubated overnight at 4 °C with anti-DJ-1 (Cell Signaling Technology, Danvers, MA, USA), anti-Beclin 1 (Cell Signaling Technology, Danvers, MA, USA), anti-Atg3 (Cell Signaling Technology, Danvers, MA, USA), anti-Atg4B (NOVUS, Saint Charles, MO, USA), anti-Atg5 (Cell Signaling Technology, Danvers, MA, USA), anti-Atg7 (Cell Signaling Technology, Danvers, MA, USA), anti-Atg16L (Cell Signaling Technology, Danvers, MA, USA), anti-LC3 (GeneTex, Irvine, CA, USA), anti-p62 (GeneTex, Irvine, CA, USA), anti-Bax (Elabscience, Houston, TX, USA), anti-Bcl-2 (Abcam, Cambridge, UK), or anti-β-actin (ProteinTech, Rosemont, IL, USA) primary antibodies diluted in TBS-T (1:1000) at 4 °C overnight. The blots were then incubated with an HRP-conjugated secondary antibody (1:20,000; Santa Cruz Biotechnology, Dallas, TX, USA) for 1 h at room temperature. Protein bands were detected using SuperSignal^TM^ West Pico PLUS Chemiluminescent Substrate (ThermoFidher Scientific, Waltham, MA, USA) and ImageQuant LAS 4000 mini biomolecular imager (GE Healthcare Life Sciences, Uppsala, Sweden). Individual protein expression was analyzed by Fusion software (VilBER, Collégien, France). IHC staining was used to analyze DJ-1 expression in paraffin-embedded sections with tissue pairs. Anti-DJ-1 antibody (1:500; Cell Signaling Technology, Danvers, MA, USA) was used for IHC staining. Tissue sections (5 μm) were dewaxed, avidin/biotin blocking, endogenous enzyme quenching, and DJ-1 staining as described previously [37]. The full uncropped Western blot images can be found in Figures S2–S6.

### 2.5. Serum DJ-1 Levels in Colorectal Cancer Patients

We used a Human Protein DJ-1 (PARK7) ELISA Kit (Cusabio, Houston, TX, USA) with a detection range between 0.235 ng/mL and 15 ng/mL to analyze serum DJ-1 expression in colorectal cancer patients. The analysis steps were according to the protocol provided by the manufacturer.

### 2.6. Cell Culture and DJ-1 Stable Knockdown

The human colorectal adenocarcinoma cell line SW620 was maintained in a humidified incubator with 5% CO_2_ at 37 °C in Dulbecco’s Modified Eagle Medium (DMEM; ThermoFisher Scientific, Waltham, MA, USA) supplemented with 10% heat-inactivated fetal bovine serum (HyClone, South Logan, UT, USA), 1% non-essential amino acids (ThermoFisher Scientific, Waltham, MA, USA), and 1% antibiotic-antimycotic (ThermoFisher Scientific, Waltham, MA, USA) as described previously [38]. Here, we used 4 vector-based short hairpin RNAs (pLKO1-shDJ-1-A, -B, -C, and -D) to knock down DJ-1 in SW620 cells. The target sequence and binding location on two *DJ-1* isoforms of 4 pLKO1-shDJ-1 plasmids are shown in Table 1. SW620 cells were transfected with pLKO1 (as vector control), pLKO1-shDJ-1-A, pLKO1-shDJ-1-B, pLKO1-shDJ-1-C, or pLKO1-shDJ-1-D by using a reduced serum medium (Opti-MEM; ThermoFisher Scientific, Waltham, MA, USA) with 0.1% lipofectamine 2000 reagent (ThermoFisher Scientific, Waltham, MA, USA). Twenty-four hours after transfection, the culture media were replaced by complete culture media with 1 μg/mL puromycin (ThermoFisher Scientific, Waltham, MA, USA). Culture media containing puromycin were changed once weekly. Six to eight weeks later, 5 stable knockdown cell clones (SW620/pLKO1, SW620/pLKO1-shDJ-1-A, SW620/pLKO1-shDJ-1-B, SW620/pLKO1-shDJ-1-C, and SW620/pLKO1-shDJ-1-D) were isolated and analyzed DJ-1 expression by Western blot.

### 2.7. MTT, Cell Migration Assay, and Colony Formation

Cell viability was detected by determining the reduction of 3-(4,5-dimethylthiazole-2yl)-2,5-diphenyltetrazolum bromide (MTT; ThermoFisher Scientific, Waltham, MA, USA). For MTT assay, 1.5 × 10^4^ cells were seeded in each well of 96-well plate overnight, then incubated with 0.5 mg/mL MTT for another 2 h. The medium was aspirated, 0.1 mL dimethylsulfoxide was added, and cell viability was measured by a microplate reader (Bio-Tek Synergy HT, BioTek Instruments, Winooski, VT, USA) at 550 nm. In order to create a standardized gap between cells, 3 × 10^4^ cells/well were seeded in each well of the 2-well silicone insert (Ibidi, Fitchburg, WI, USA) for 24 h. The cell inserts were removed for cell migration, and images were taken 24 h later for migration distance analysis. For colony formation, 500 cells/6-well were seeded for 14 days. After 14 days, colonies were fixed with methanol and stained with crystal violet to evaluate colony formation.

### 2.8. ATP Production, Intracellular Reactive Oxygen Species (ROS) Detection, Mitochondrial Depolarization Analysis, and Immunofluorescence Staining

For ATP production, 8 × 10^5^ cells/6-well were seeded overnight. ATP production of each cell clone was measured by an ATP colorimetric assay kit (BioVision, San Francisco, CA, USA) in a microplate reader (Bio-Tek Synergy HT, BioTek Instruments, Winooski, VT, USA) at A570 according to the protocol provided by the manufacturer. For intracellular ROS detection and mitochondrial membrane potential analysis, 3 × 10^4^ cells/96-well were seeded overnight. Here, we used DCFDA (2′,7′-Dichlorodihydrofluorescein diacetate; Sigma-Aldrich, Saint Louis, MO, USA) to detect intracellular ROS in each cell clone described previously [39]. Changes in mitochondrial depolarization were measured by JC-10^TM^ dye (ATT Bioquest, Sunnyvale, CA, USA) using a florescence microplate reader (Bio-Tek Synergy HT, BioTek Instruments, Winooski, VT, USA) as described previously [36]. For labeling of the autophagosomes and mitochondria, cells were seeded on coverslips overnight and then fixed, blocked, permeabilized, stained with antibodies against LC3 (1:500; GeneTex Irvine, CA, USA) and EF-Tu (1:500; Santa Cruz Biotechnology, Dallas, TX, USA), and then with Alexa-488-conjugated goat anti-rabbit secondary antibody (1:1000; Thermo Fisher Scientific, Waltham, MA, USA) and Alexa-543-conjugated goat anti-mouse secondary antibody (1:1000; Thermo Fisher Scientific, Waltham, MA, USA) as described previously [36]. Confocal images were obtained using excitation wavelengths of 488 nm (for Alexa-488) or 543 nm (for Alexa-543) by Leica confocal microscope (TCS SP8, Leica, Wetzlar, Germany) as described previously [36].

### 2.9. Statistics

All data analyses were performed using GraphPad Prism 5 software (GraphPad Software, San Diego, CA, USA). Quantitative results are expressed as the mean ± standard error of the mean (S.E.M.). Significant differences between two independent groups (microarray and Western blot data of Figure 5) were determined by the Student’s *t*-test. The other data were analyzed by one-way analysis of variance (ANOVA) followed by Newman–Keuls multiple comparison testing to compare the between-group statistical significance. A *p*-value of < 0.05 was considered to be statistically significant.

## 3. Results

### 3.1. DJ-1 mRNA Overexpression in Tumor Tissues (T) but Not Adjacent Normal Tissues (N) Is Positively Correlated with TNM Stages of Colorectal Cancer Patients, and DJ-1′s Function Might Associate with Autophagy/Mitophagy

**Figure 1:**
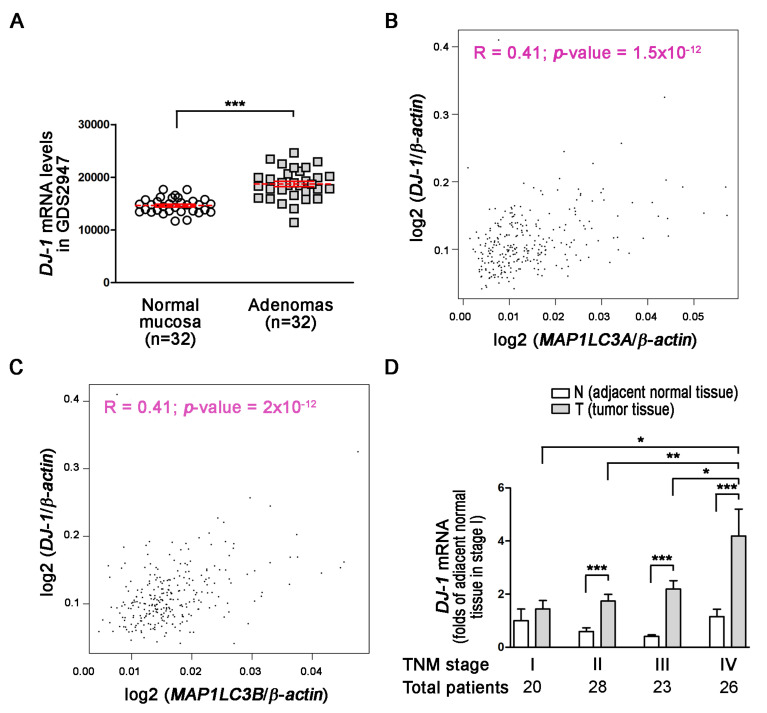
*DJ-1* mRNA overexpression in tumor tissues (T) but not adjacent normal tissues (N) is positively correlated with TNM stages of colorectal cancer patients, and *DJ-1′s* function may associate with autophagy/mitophagy. (**A**) The microarray analysis result of the GSD2947 dataset shows that *DJ-1* mRNA levels were higher in colorectal adenoma than normal mucosa in 32 pairs of tissues from 28 colorectal cancer patients of Belcolle City Hospital (Viterbo, Italy). Data are expressed as the means ± S.E.M; the Student’s *t*-test determined the *p*-value. ***: *p* < 0.001 compared with normal mucosa. (**B**) The analysis result of GEPIA (Gene Expression Profiling Interactive Analysis) shows *DJ-1* mRNA expression was positively corrected with *MAP1LC3A* mRNA expression (R = 0.41). (**C**) The analysis result of GEPIA shows *DJ-1* mRNA expression was positively corrected with *MAP1LC3B* mRNA expression (R = 0.41). The Pearson correlation coefficients determined the correlation of two gene expressions. (**D**) The real-time quantitative PCR result of 20 pairs of tissues from Taiwanese patients with TNM stage I colorectal cancer shows that *DJ-1* mRNA did not significantly change between adjacent normal tissues (N) and tumor tissues (T). In tissue pairs of TNM stage II (*n* = 28), III (*n* = 23), and IV (*n* = 26) colorectal cancer patients, *DJ-1* mRNA significantly increased in T than N. In addition, the increase of *DJ-1* mRNA showed TNM stage-dependent. It indicates *DJ-1* mRNA overexpression in tumor tissues is positively correlated with TNM stages of colorectal cancer patients. Data are expressed as the means ± S.E.M; the *p*-value was determined by one-way ANOVA/Newman-Keuls test. *: *p* < 0.05, **: *p* < 0.01, ***: *p* < 0.001 compared with the respectively group.

DJ-1 is a neuroprotective and mitophagy protein against reactive oxygen species in Parkinson’s disease [36]. However, the role of DJ-1 in colorectal cancer remains elusive. First, we used the microarray data of the GSD2947 dataset to analyze *DJ-1* mRNA levels in 32 pairs of adenoma tissues and normal mucosa tissues (≥2 cm from the side of the polyp), which were obtained from the same colon segment from 28 colorectal cancer patients. The microarray result shows that *DJ-1* mRNA levels increased in 32 colorectal adenoma tissues than respectively paired normal mucosa tissues in the patients of the Belcolle City Hospital (Viterbo, Italy) (Figure 1A; *p* < 0.001). During autophagy/mitophagy activation, LC3A and LC3B are involved in the formation of autophagosomal vacuoles. *MAP1LC3A* and *MAP1LC3B* are protein-coding genes of LC3A and LC3B, respectively. The analysis results of GEPIA (Gene Expression Profiling Interactive Analysis) show *DJ-1* mRNA expression was positively corrected with *MAP1LC3A* and *MAP1LC3B* expressions in tumor tissues of subjects with colon adenocarcinoma (R = 0.41 in both two; Figure 1B,C). It suggests that *DJ-1* expression in colon adenocarcinoma might associate with autophagy/mitophagy. We then collected specimens and used real-time quantitative PCR to analyze *DJ-1* mRNA expressions in 97 tissue pairs from Taiwanese patients with different TNM stages of colorectal cancer. In 20 paired adjacent normal tissues (N) and tumor tissues (T) obtained from 20 patients with TNM stage I colorectal cancer, *DJ-1* did not significantly change between N and T (Figure 1D). In the tissue pairs from patients with TNM stage II (*n* = 28), III (*n* = 23), and IV (*n* = 26) colorectal cancer, *DJ-1* significantly increased in T than N (Figure 1D). In addition, the increase of *DJ-1* in T was TNM stage-dependently (Figure 1D). Our data indicate that *DJ-1* mRNA overexpression in tumor tissues is positively correlated with TNM stages of colorectal cancer patients, and its function might associate with autophagy/mitophagy.

### 3.2. DJ-1 Protein Overexpression in Colorectal Tumor Tissues (T) but Not Adjacent Normal Tissues (N) Is Positively Correlated with TNM Stages of Colorectal Cancer Patients

**Figure 2:**
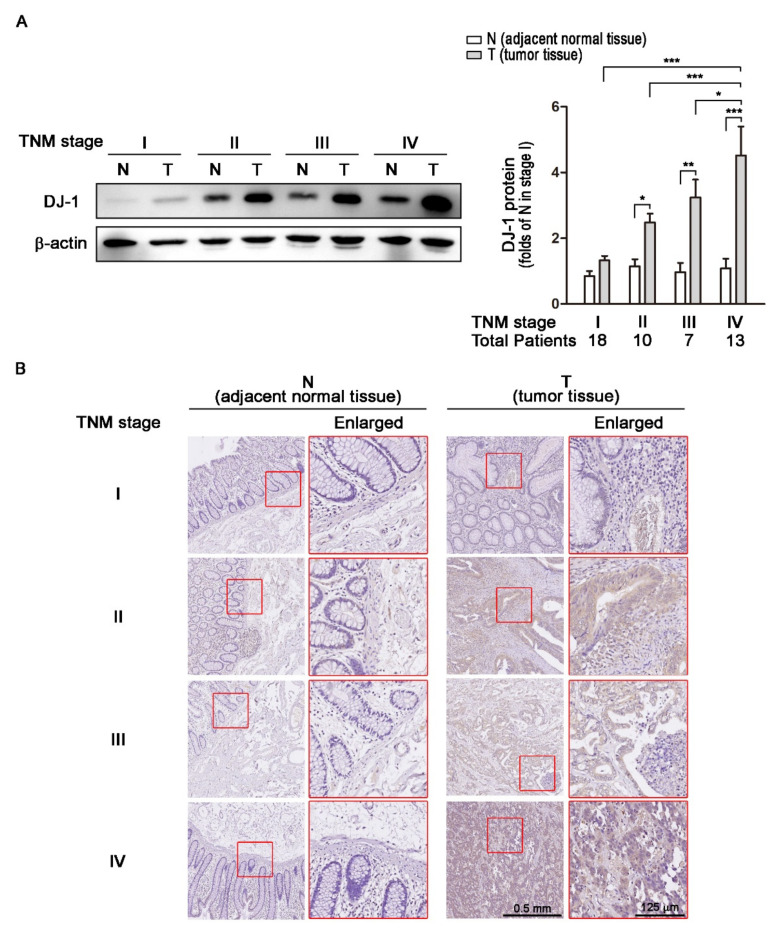
DJ-1 protein overexpression in tumor tissues is positively correlated with TNM stages of colorectal cancer patients. (**A**) The Western blot result of DJ-1 protein expressions in tissue pairs from 18 Taiwanese patients with TNM stage I colorectal cancer shows that DJ-1 did not significantly change between tumor tissue (T) and adjacent normal tissue (N). In the tissue pairs from TNM stage II (*n* = 10), III (*n* = 7), and IV (*n* = 13) colorectal cancer patients, DJ-1 proteins significantly increased in T but not N. In addition, the increase of DJ-1 protein was TNM stage-dependent. The quantification data of DJ-1 protein levels are shown on the right panel. (**B**) DJ-1 immunohistochemistry (IHC) data show that DJ-1 staining increased in T but not N in Taiwanese patients with colorectal cancer that was positively correlated with TNM stages. The enlarged images on the right panels are from the respective red squares of the left panels. Our data suggest that DJ-1 protein increase in tumor tissue is positively correlated with TNM stages of colorectal cancer patients. Quantitative data are expressed as the means ± S.E.M; the *p*-value was determined by one-way ANOVA/Newman-Keuls test. *: *p* < 0.05, **: *p* < 0.01, ***: *p* < 0.001 compared with the respectively group.

Transcription and RNA processing regulate mRNA expressions; translation and post-transcriptional regulation control protein expressions. Since *DJ-1* mRNA overexpression in tumor tissues was positively correlated with TNM stages, we further analyzed DJ-1 protein levels. We used Western blot to analyze DJ-1 expressions in 48 tissue pairs from Taiwanese patients with different TNM stages of colorectal cancer. In 18 paired adjacent normal tissues (N) and tumor tissues (T) obtained from 18 patients with TNM stage I colorectal cancer, DJ-1 did not significantly change (Figure 2A). In TNM stage II (*n* = 10), III (*n* = 7), and IV (*n* = 13) colorectal cancer patients, DJ-1 significantly increased only in T but not N, and the increase of DJ-1 in T was TNM stage-dependent (Figure 2A). The quantification data of DJ-1 protein levels are shown on the right panel of Figure 2A. DJ-1 immunohistochemistry (IHC) data also show that DJ-1 staining increase only appeared in T but not N in each stage, and DJ-1 staining in T increased with TNM stages (Figure 2B). The enlarged images on the right panels are from the respective red squares of the left panels (Figure 2B). Our data suggest that DJ-1 protein overexpression appears in tumor tissues but not adjacent normal tissues, and DJ-1 protein overexpression in tumor tissues is positively correlated with TNM stages of colorectal cancer patients.

### 3.3. Increases in Serum DJ-1 Are Positively Correlated with TNM Stages of Colorectal Cancer in Taiwanese Patients

**Figure 3:**
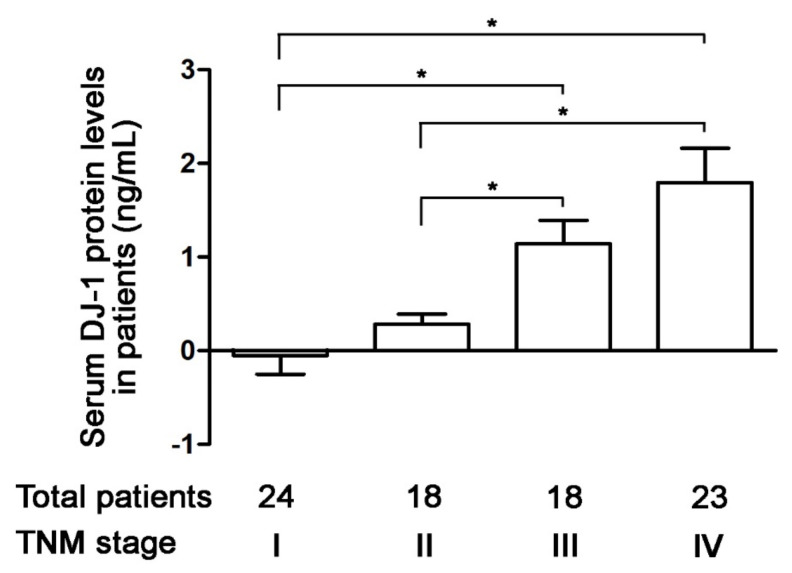
Increases in serum DJ-1 level are positively correlated with TNM stages of colorectal cancer in Taiwanese patients. The ELISA data show that serum DJ-1 levels in 24 patients with TNM stage I colorectal cancer were about zero. Serum DJ-1 protein levels in TNM stage II (*n* = 18), III (*n* = 18), and IV (*n* = 23) colorectal cancer patients show TNM stage-dependently increase. It suggests that serum DJ-1 levels are positively correlated with the TNM stage of colorectal cancer in Taiwanese patients, which is consistent with our *DJ-1* mRNA and DJ-1 protein expressions in tumor tissues. It indicates that DJ-1 is a colorectal cancer biomarker that increases in both tumor tissues and serum. Data are expressed as the means ± S.E.M, and the *p*-value was determined by one-way ANOVA/Newman-Keuls test. *: *p* < 0.05 compared with the respective group.

Serum DJ-1 is a diagnostic marker and prognostic factor for pancreatic cancer [30]. In 2020, Wang et al. used tissue microarray from patients to demonstrate DJ-1 expression in tumor tissues is a new prognostic marker in colorectal cancer [28]. Since our data of tumor tissues are consistent with Wang et al., we further analyzed the correction of serum DJ-1 levels and TNM stages of colorectal cancer in 83 Taiwanese patients. Our ELISA data show that serum DJ-1 levels in 24 patients with TNM stage I colorectal cancer were about zero (Figure 3). Serum DJ-1 levels in TNM stage II (*n* = 18), III (*n* = 18), and IV (*n* = 23) colorectal cancer patients showed TNM stage-dependently increase (Figure 3, *p* < 0.05). This indicates that serum DJ-1 levels in colorectal cancer patients are TNM stage-dependent, consistent with our data of *DJ-1* mRNA and DJ-1 protein expressions in tumor tissues. Our data suggest that DJ-1 is a colorectal cancer biomarker. In addition, DJ-1 increase in both tumor tissues and serum is positively correlated with TNM stages of colorectal cancer.

### 3.4. DJ-1 Knockdown Inhibits Cell Survival, Cell Migration, and Colony Formation in Metastatic Colorectal Adenocarcinoma Cells

**Figure 4:**
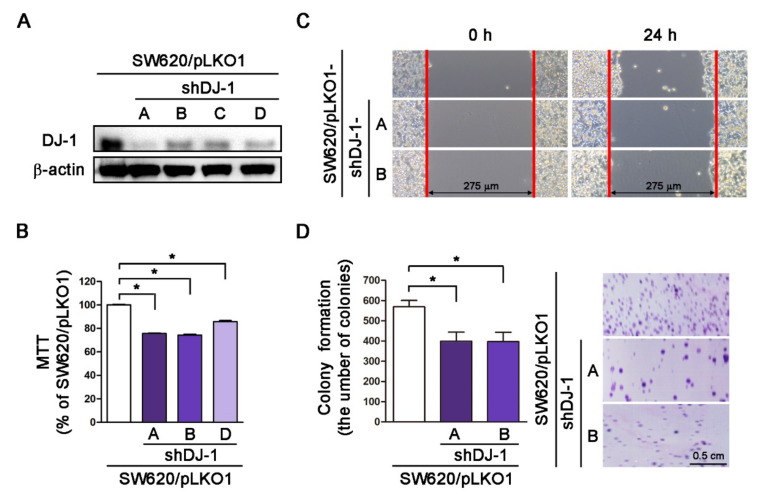
DJ-1 knockdown inhibits cell survival, cell migration, and colony formation in metastatic colorectal adenocarcinoma cells. (**A**) The Western blot result shows that pLKO-1-shDJ-1 plasmids effetely inhibited DJ-1 expression in four SW620 stable knockdown cell clones (SW620/pLKO1-shDJ-1-A, -B, -C, and -D) then the empty vector-transfected control (SW620/pLKO1). (**B**) The cell viability result by MTT assay shows that three DJ-1 knockdown cell clones (SW620/pLKO1-shDJ-1-A, -B, and -D) reduced cell viability than control (SW620/pLKO1). (**C**) Twenty-four hours after standardized gaps (275 μm) were created for cell migration between cells by 2-well cell insert, SW620/pLKO1-shDJ-1-A and -B showed slower cell migration than control. (**D**) The result of adhesion-independent cell proliferation by cancer cell colony formation shows that SW620/pLKO1-shDJ-1-A and -B were lower in colony numbers than control after 500 cells/6-well were seeded for 14 days. The images of the colonies stained with crystal violet are shown on the right panels. Quantitative data are expressed as the means ± S.E.M; the *p*-value was determined by one-way ANOVA/Newman-Keuls test. *: *p* < 0.05 compared with the control group.

Our microarray data in Figure 1A shows *DJ-1* increased in adenoma than normal mucosa tissues. Additionally, *DJ-1* mRNA and DJ-1 protein increased in TNM stage II, III, and IV colorectal cancer tumor tissues but not TNM stage I. SW620 is a metastasis-derived human colorectal adenocarcinoma cell; SW620 shows higher tumorigenic and metastatic than SW480 cells [40]. Zhou et al. showed that DJ-1 increased more significantly in SW620 than SW480 cells [26]. We then transfected four vector-based *DJ-1*-specific short hairpin RNAs (pLKO1-shDJ-1-A, -B, -C, and -D) into SW620 cells and used puromycin to select DJ-1 stable knockdown cell clones. Western blot result of Figure 4A shows that the four pLKO1-shDJ-1 plasmids (pLKO1-shDJ-1-A, -B, -C, and -D) effectively reduced DJ-1 expressions in all four stable knockdown cell clones (SW620/pLKO1-shDJ-1-A, -B, -C, and -D) as compared with the empty vector-transfected control (SW620/pLKO1). We further evaluated cell viability, cell migration, and colony formation in DJ-1 knockdown cell clones. The cell viability data by MTT assay show that three DJ-1 knockdown cell clones (SW620/pLKO1-shDJ-1-A, -B, and -D) had lower cell viability than control (SW620/pLKO1) (Figure 4B, *p* < 0.05). Cell migration is a highly integrated, multi-step process that plays an essential role in cancer progression. Twenty-four hours after a standardized gap (275 μm) was created for cell migration between cells by 2-well cell insert, SW620/pLKO1-shDJ-1-A and -B showed slower cell migration than control (Figure 4C). The adhesion-independent cell proliferation data by colony formation show SW620/pLKO1-shDJ-1-A and -B were lower in colony numbers than control after 500 cells were seeded for 14 days (Figure 4D, *p* < 0.05). The images of the colonies stained with crystal violet are shown on the right panels of Figure 4D. These data indicate that DJ-1 knockdown inhibits cell viability, cell migration, and colony formation in metastatic colorectal adenocarcinoma cells.

### 3.5. DJ-1 Knockdown Induces an Incomplete Autophagic Response and Cell Apoptosis in Metastatic Colorectal Adenocarcinoma Cells

**Figure 5:**
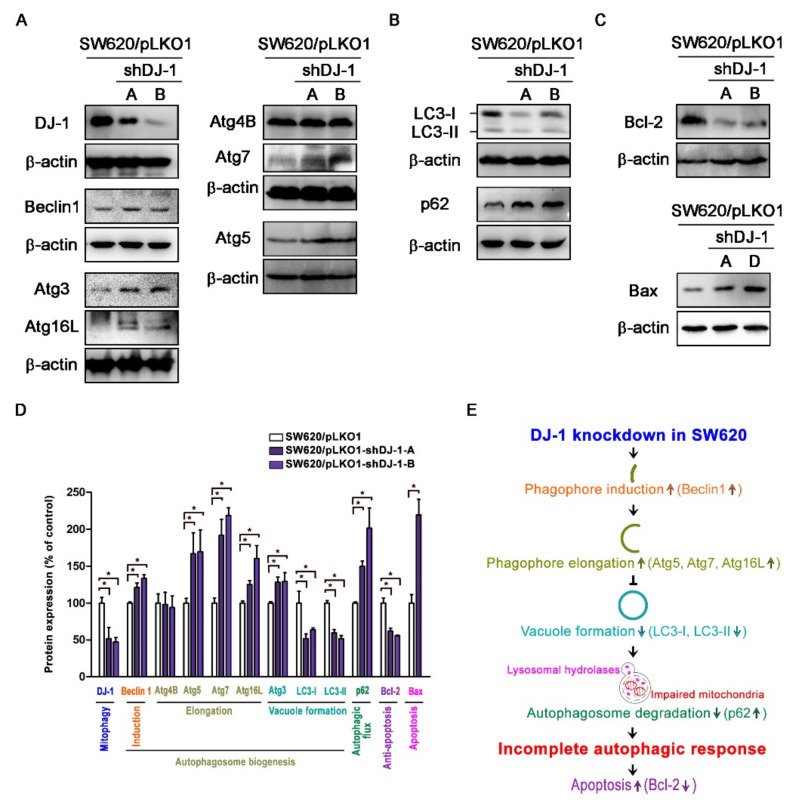
DJ-1 knockdown induces an incomplete autophagic response and cell apoptosis in metastatic colorectal adenocarcinoma cells SW620. (**A**) Western blot results show that pLKO-1-shDJ-1 plasmids effectively inhibited DJ-1 expressions in SW620/pLKO1-shDJ-1-A and -B cell clones compared with the control cell clone (SW620/pLKO1). Beclin 1, Atg3, Atg5, Atg7, and Atg16L increased in DJ-1 knockdown cell clones (SW620/pLKO1-shDJ-1-A and -B) compared with control (SW620/pLKO1). It indicates that DJ-1 knockdown enhances phagophore induction (Beclin1 increased) and elongation (Atg5, Atg7, and Atg16L increased) during autophagosome biogenesis in metastatic colorectal adenocarcinoma cells. (**B**) Western blot results show LC3 decreased and p62 increased in DJ-1 stable knockdown cell clones (SW620/pLKO1-shDJ-1-A and -B) compared with control (SW620/pLKO1), indicating that DJ-1 knockdown inhibits vacuole formation (LC3-I and LC3-II decreased) and autophagosome degradation (p62 increased) in metastatic colorectal adenocarcinoma cells during autophagosome biogenesis. (**C**) Western blot results show that Bcl-2 decreased and Bax increased in DJ-1 knockdown cell clones (SW620/pLKO1-shDJ-1-A, -B, or -D) compared with control (SW620/pLKO1). Our data suggest that DJ-1 knockdown induced an incomplete autophagy response and cell apoptosis. (**D**) The quantification data of each protein level with its function are shown here. Data are expressed as the means ± S.E.M; the Student’s *t*-test determined the *p*-value. *: *p* < 0.05 compared with control (SW620/pLKO1). (**E**) A proposed model shows that DJ-1 knockdown induces incomplete autophagy.

Our microarray data (Figure 1B,C) show *DJ-1* mRNA expression was positively correlated with *MAP1LC3A* and *MAP1LC3B* expressions in tumor tissues from subjects with colon adenocarcinoma. It suggests that *DJ-1* expression in colon adenocarcinoma might associate with autophagy/mitophagy. The process of autophagy includes autophagy induction, substrate recognition and selection, autophagosome biogenesis (phagophore nucleation/induction, phagophore elongation, substrate binding, and vacuole formation), autophagosome-lysosome fusion, and substrate degradation and recycling [6,7]. Since the function of *DJ-1* might associate with autophagy/mitophagy, we analyzed autophagosome biogenesis in DJ-1 stable knockdown cells. Western blot data of Figure 5A show DJ-1 decreases in DJ-1 stable knockdown cell clones (SW620/pLKO1-shDJ-1-A and -B) more than control (SW620/pLKO1). DJ-1 knockdown cell clones (SW620/pLKO1-shDJ-1-A and -B) showed higher Beclin 1, Atg3, Atg5, Atg7, and Atg16L than control (Figure 5A). It suggests DJ-1 knockdown enhances phagophore induction (Beclin1 increased) and elongation (Atg5, Atg7, and Atg16L increased) during autophagosome biogenesis in metastatic colorectal adenocarcinoma cells. We found LC3 decreased and p62 increased in DJ-1 knockdown cell clones (SW620/pLKO1-shDJ-1-A and -B) compared with control (Figure 5B). It indicates that DJ-1 knockdown represses vacuole formation (LC3-I and LC3-II decreased) and autophagosome degradation (p62 increased) during autophagosome biogenesis. We also found Bcl-2 decreased and Bax increased in DJ-1 knockdown cells (SW620/pLKO1-shDJ-1-A, -B, or -D) compared with control (Figure 5C). Figure 5D is the quantification data of each protein level. Our data suggest that DJ-1 knockdown induced an incomplete autophagic response and cell apoptosis in metastatic colorectal adenocarcinoma cells. A proposed model of DJ-1 knockdown-induced incomplete autophagy is shown in Figure 5E.

### 3.6. DJ-1 Knockdown Has No Impact on Adenosine Triphosphate Production; DJ-1 Knockdown Enhances Intracellular Reactive Oxygen Species Generation, Damaged Mitochondrial Accumulation and Mitophagy Inhibition in Metastatic Colorectal Adenocarcinoma Cells

**Figure 6:**
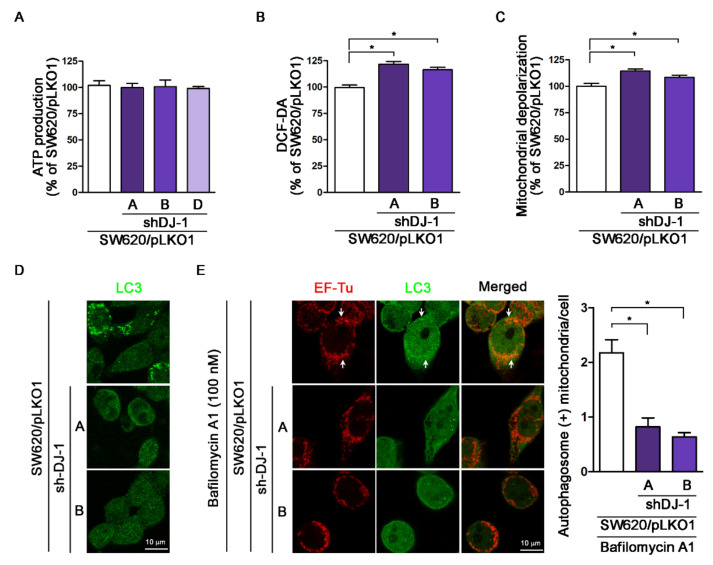
DJ-1 knockdown does not affect adenosine triphosphate (ATP) production; DJ-1 knockdown enhances intracellular reactive oxygen species (ROS) generation, damaged mitochondrial accumulation and mitophagy inhibition in metastatic colorectal adenocarcinoma cells. (**A**) The ATP production result shows that DJ-1 knockdown did not impact ATP production in three DJ-1 stable knockdown cell clones (SW620/pLKO1-shDJ-1-A, -B, and -D) as compared with control (SW620/pLKO1). (**B**) The DCF-DA assay result shows that DJ-1 knockdown cell clones (SW620/pLKO1-shDJ-1-A and -B) had higher intracellular ROS than control. (**C**) DJ-1 knockdown cell clones (SW620/pLKO1-shDJ-1-A and -B) showed increased mitochondrial depolarization than control. (**D**) For mitophagy analysis, we used anti-EF-Tu and anti-LC3 antibodies to stain mitochondria and autophagosomes, respectively. Images of LC3 immunofluorescence staining obtained from confocal microscopy show that control (SW620/pLKO1) cells contained more autophagosomes (LC3 punctate dots) than DJ-1 knockdown cell clones (SW620/pLKO1-shDJ-1-A and -B). (**E**) Images of immunofluorescence double staining obtained from confocal microscopy show mitochondria colocalized with autophagosomes in control (SW620/pLKO1). Arrows indicate the colocalizations of EF-Tu and LC3. DJ-1 knockdown cell clones (SW620/pLKO1-shDJ-1-A and -B) inhibited mitochondria colocalization with autophagosomes. These results indicate that DJ-1 knockdown does not influence mitochondrial ATP production but enhances intracellular ROS generation and damaged mitochondria accumulation and inhibits mitochondria colocalization with autophagosomes. The quantification data of autophagosome-positive mitochondria per cell are shown on the right panel. Data are expressed as the means ± S.E.M; the *p*-value was determined by one-way ANOVA/Newman–Keuls test. * *p* < 0.05 compared with the control group.

Mitophagy is the selective autophagic degradation of mitochondria [41]. Originally, DJ-1 is linked with Parkinson’s disease with antioxidant functions [42]. Our previous study demonstrates that DJ-1 controls mitochondrial quality by activating mitophagy to remove damaged mitochondria against neuronal apoptosis [36]. Loss of DJ-1 leads to loss of mitochondrial polarization, fragmentation of mitochondria, and accumulation of markers of autophagy (LC3 puncta and lipidation) around mitochondria in human dopaminergic cells [19]. During adenosine triphosphate (ATP) synthesis in mitochondria, the mitochondrial respiratory chain produces reactive oxygen species (ROS) that cause mitochondrial oxidative damage, which contributes to mitochondrial dysfunction and cell apoptosis [43]. For analysis of mitochondrial functions in DJ-1 knockdown cell clones, we evaluated ATP production, intracellular ROS status, and mitochondrial depolarization. Figure 6A shows that ATP production did not differ in three DJ-1 knockdown cell clones (SW620/pLKO1-shDJ-1-A, -B, and -D) compared with control (SW620/pLKO1). The DCF-DA assay result shows that DJ-1 knockdown cell clones (SW620/pLKO1-shDJ-1-A and -B) had higher intracellular ROS than control (Figure 6B). Additionally, DJ-1 knockdown cell clones (SW620/pLKO1-shDJ-1-A and -B) increased mitochondrial membrane depolarization than control (Figure 6C). For mitophagy analysis, we used anti-EF-Tu and anti-LC3 antibodies to stain mitochondria and autophagosomes, respectively. Images of LC3 immunofluorescence staining obtained from confocal microscopy show that control (SW620/pLKO1) cells contained more autophagosomes (LC3 punctate dots) than DJ-1 knockdown cell clones (SW620/pLKO1-shDJ-1-A and -B) (Figure 6D). After cells were treated with bafilomycin A1 for 4 h to prevent autophagosome-lysosome fusion, mitophagy was detected by mitochondria and autophagosome colocalization. Images of immunofluorescence double staining show mitochondria colocalized with autophagosomes in control cells (SW620/pLKO1) (Figure 6E). Arrows indicate the colocalizations of EF-Tu and LC3. DJ-1 knockdown cell clones (SW620/pLKO1-shDJ-1-A and -B) inhibited mitochondria colocalization with autophagosomes (Figure 6E). The quantification data of autophagosome-positive mitochondria per cell are shown on the right panel. These results demonstrate DJ-1 knockdown does not influence mitochondrial ATP production but enhances intracellular ROS generation, damaged mitochondria accumulation, and mitophagy inhibition. It suggests the function of DJ-1 is associated with mitophagy to remove dysfunctional or damaged mitochondria in metastatic colorectal adenocarcinoma cells.

## 4. Discussion

**Figure 7:**
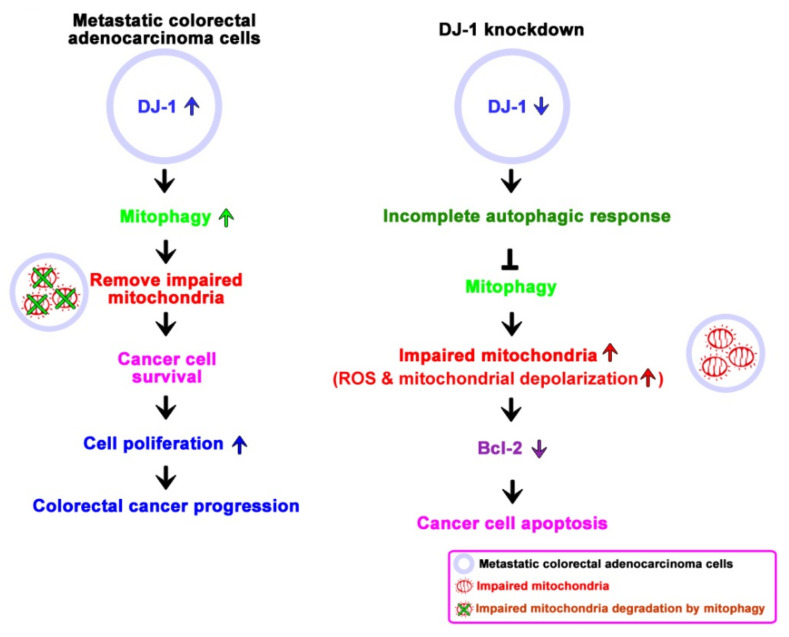
A proposed model shows the role of DJ-1 activates mitophagy to promote colorectal cancer progression. In metastatic colorectal adenocarcinoma cells SW620, DJ-1 activates mitophagy to remove dysfunctional mitochondria against tumor cell apoptosis. DJ-1 upregulation enhances cell survival and proliferation in metastatic colorectal adenocarcinoma cells, resulting in colorectal cancer progression. Inhibition of DJ-1 expression by DJ-1 knockdown induces an incomplete autophagic response and mitophagy inhibition that causes metastatic colorectal adenocarcinoma cells unable to remove damaged mitochondria and further induce cancer cell apoptosis.

Colorectal cancer is one of the most common types of cancer worldwide, and it is often recognized too late for successful therapy [44]. In the present study, we used cancer cells and patients’ tissues and serum to demonstrate the clinical value and physiological function of DJ-1 in colorectal cancer. We found *DJ-1* mRNAs and proteins overexpression in TNM stage II, III, and IV but not stage I tumor tissues (T) than adjacent normal tissues (N) in Taiwanese patients with colorectal cancer. Moreover, DJ-1 levels in both patients’ tumor tissues and serum positively correlated with the TNM stage in Taiwanese patients. Serum DJ-1 might be clinically valuable as a non-invasive cancer marker for colorectal cancer prognosis in Taiwanese patients. Wang et al. (2020) showed that SW620 cells express the highest DJ-1 than FHC, DLD-1, RKO, HCT116, SW480, HT-29, and HCT15 cells [28]. Zhou et al. (2018) showed that DJ-1 significantly increased in SW620 than SW480, HT29, and HCT116 cells [26]. SW620 is a metastasis-derived human colorectal adenocarcinoma cell; SW620 shows higher tumorigenic and metastatic properties than SW480 cells [40]. Since DJ-1 expression is positively correlated with the TNM stage in the present study, we performed DJ-1 knockdown in SW620 cells. By manipulating DJ-1 expression in metastatic colorectal adenocarcinoma cells SW620, we found that DJ-1 stable knockdown inhibits cell survival, migration, and colony formation. DJ-1 knockdown induces an incomplete autophagic response and cell apoptosis in SW620. Additionally, DJ-1 stable knockdown in SW620 does not affect ATP production; DJ-1 knockdown enhances intracellular reactive oxygen species (ROS) generation, damages mitochondrial accumulation and mitophagy inhibition. Our data suggest that DJ-1 activates mitophagy to remove dysfunctional mitochondria against tumor cell apoptosis. In metastatic colorectal adenocarcinoma cells, DJ-1 overexpression enhances cell survival and proliferation to promote colorectal cancer progression. Inhibition of DJ-1 expression by DJ-1 knockdown induces an incomplete autophagic response and mitophagy inhibition that cause metastatic colorectal adenocarcinoma cells cannot remove damaged mitochondria and further induce cancer cell apoptosis. Figure 7 is a proposed model that shows the role of DJ-1-induced mitophagy in colorectal cancer progression.

DJ-1 overexpression is found in malignant tissues of ovarian carcinoma, ductal carcinoma of the breast, pancreatic cancer, and colorectal cancer [22,23,26,27,28,30]. Zheng et al. (2018) showed that DJ-1 promotes colon cancer cell survival under hypoxia by modulating HIF-1α expression through the PI3K-AKT pathway [27]. They found DJ-1 expressed in 50 out of 73 (68.5%) colorectal cancer specimens, and nuclear DJ-1 expression was strong in tumor cells but relatively lower in normal epithelial cells [27]. Lin et al. (2017) demonstrated DJ-1 overexpression activated protein kinase Akt and downregulated tumor suppressor PTEN in colon cancer cell lines HCT116 and SW480, whereas DJ-1 knockdown upregulated PTEN expression and effectively suppressed colon cancer cell invasion and proliferation [24]. Our study results show that *DJ-1* mRNA increased in patients’ tumor tissues positively correlated with the TNM stage of colorectal cancer. Additionally, DJ-1 protein increased in both tumor tissues and serum from patients with colorectal cancer that were positively correlated with the TNM stage of colorectal cancer. It indicates the clinical value of DJ-1 in colorectal cancer diagnosis.

Activated autophagy is tumor-suppressing (anti-tumor) during the early stages of tumorigenesis, but reduced or incomplete autophagy is found in tumor cells associated with malignant transformation [45]. Mitophagy is the selective engulfment and clearance of damaged or excessive mitochondria by autophagy; defective mitophagy contributes to breast cancer progression [13]. Mitochondria are not only the powerhouse of cells but also trigger death-promoting signaling cascades for programmed cell death. Removing damaged mitochondria through mitophagy requires two steps: induction of general autophagy and priming of damaged mitochondria for selective autophagic recognition [46]. Mitophagy inhibition in melanoma, lung cancer, and pancreatic cancer may be a viable strategy to sensitive these tumors to therapeutics [13]. The present study found that DJ-1 knockdown enhanced intracellular reactive oxygen species (ROS) generation, damaged mitochondrial accumulation, incomplete autophagic response, and cell apoptosis (Bcl-2 decreased and Bax increased) in metastatic colorectal adenocarcinoma cells SW620. It suggests that mitophagy inhibition by DJ-1 knockdown is an anti-cancer strategy for treating metastatic colorectal cancer.

Colorectal cancer screening requires tools and methods with high sensitivity and specificity. Furthermore, they must be safe, cheap, and widely accepted by patients. Collecting blood, urine, saliva, sputum, and stool specimens are safer and convenient than solid tumor tissue, bone marrow, lymph node, ascites, and cerebrospinal fluid specimens. Several blood markers are used for the diagnosis and prognosis of colorectal cancer, e.g., carcinoembryonic antigen (CEA), carbohydrate antigen (CA 19.9), tissue polypeptide specific antigen (TPS), tumor-associated glycoprotein-72 (TAG-72), hematopoietic growth factors (HGFs), macrophage-colony stimulating factor (M-CSF), granulocyte-macrophage-colony stimulating factor (GM-CSF), interleukin-3, interleukin-6, alcohol dehydrogenase, and lysosomal exoglycosidases, but none of these tests have excellent diagnostic accuracy and all of that with significant limitations [44]. In Parkinson’s disease, DJ-1 protein levels are elevated in the cerebrospinal fluid and plasma of sporadic Parkinson’s disease patients as a biomarker [33]. In pancreatic cancer, serum DJ-1 is a diagnostic marker and prognostic factor [30]. He et al. (2011) showed that the median (the range) of serum DJ-1 levels in healthy subjects (*n* = 40), patients with chronic pancreatitis (*n* = 43) and pancreatic cancer (*n* = 47) were 0.6236 (0.4221–2.0000) ng/mL, 1.26 (0.3750–2.3250) ng/mL, and 2.041 (0.8000–8.000) ng/mL, respectively [30]. In addition, higher serum DJ-1 was correlated with shorter overall survival in pancreatic cancer [30]. In the present study, the median (the range) of serum DJ-1 levels in colorectal cancer patients with TNM stage I (*n* = 24), II (*n* = 18), III (*n* = 18), and IV (*n* = 23) were −0.2938 (−1.2017–3.6186) ng/mL, 0.2996 (−0.3035–1.4701) ng/mL, 0.8508 (−0.3943–3.5064) ng/mL, and 1.1621 (0.2153–7.0042) ng/mL, respectively. Our serum DJ-1 range in patients with stage IV colorectal cancer is similar to patients with pancreatic cancer. Our data indicate that serum DJ-1 levels are positively correlated with TNM stages of colorectal cancer. To our knowledge, this is the first report to show serum DJ-1 has the potential as a serum tumor biomarker for colorectal cancer diagnosis or monitoring. Further studies are needed to provide the sensitivity and specificity data of serum DJ-1 in colorectal cancer diagnosis and prognosis.

There are still some limitations in the present study. Firstly, our sample size to detect DJ-1 expression in the tumor tissues and serum of Taiwanese patients with colorectal cancer was small (*n* = 97 and 83, respectively); therefore, more samples are needed to support our results that the high expression of DJ-1 could predict the TNM stage of colorectal cancer in Taiwanese patients. Secondly, we did not compare serum DJ-1 and other conventional biomarkers of colorectal cancer (e.g., CEA, CA 19.9, and TAG-72) in the present study. We need further studies and more serum samples to provide the sensitivity and specificity data for DJ-1 in colorectal cancer diagnosis.

Colorectal cancer is one of the most common cancer types worldwide; it is the second most common cancer and the third most common cancer-associated death in Taiwan. None of the serum markers for colorectal cancer screening has excellent diagnostic accuracy. About 22% of colorectal cancer patients are metastatic at initial diagnosis, and the patients with metastatic colorectal cancer face a poor prognosis and have a relative 5-year survival rate of 14% [4]. The present study found that DJ-1 activates mitophagy to remove dysfunctional mitochondria against metastatic colorectal adenocarcinoma cell apoptosis. Additionally, DJ-1 knockdown in metastatic colorectal adenocarcinoma induces an incomplete autophagic response and cancer cell apoptosis. It indicates that DJ-1-induced mitophagy promotes colorectal cancer progression. Our study is the first report demonstrating that DJ-1 might be clinically valuable as serum and tissue biomarkers for predicting the TNM stage in colorectal cancer patients. Since DJ-1-induced mitophagy promotes tumor progression, DJ-1 inhibition is a potential therapeutic strategy for colorectal cancer treatment.

## 5. Conclusions

In summary, we reported for the first time to our knowledge that DJ-1 might be clinically valuable as serum and tissue biomarkers for predicting the TNM stage in colorectal cancer patients. In addition, DJ-1 knockdown inhibits mitophagy that causes metastatic colorectal adenocarcinoma cells unable to remove damaged mitochondria and further enhance cancer cell apoptosis. Since DJ-1-induced mitophagy promotes tumor progression, DJ-1 inhibition is a potential therapeutic strategy for colorectal cancer treatment.

## Figures and Tables

**Table 1:** Primer and probe sequences used for RT-qPCR assays; the target sequence and binding site on two *DJ-1* isoforms of four shDJ-1 plasmids used in the study.

Gene	Forward Primer	Reverse Primer	Probe
*DJ-1*	GATGTCATGAGGCGAGCTG	TGACCACATCACGGCTACAC	CCTGGAGC
*β-actin*	ATTGGCAATGAGCGGTTC	CGTGGATGCCACAGGACT	CTTCCAGC
**Vector-Based shDJ-1 Plasmid**	**Target Sequence on *DJ-1***	**Binding Site on the *DJ-1* Isoform**	
		**NM_007262.5**	**NM_001123377.2**
pLKO1-shDJ-1-A	ACTTAGAGAAACAGGCCGTTA	697–717	639–659
pLKO1-shDJ-1-B	GCAATTGTTGAAGCCCTGAAT	605–625	547–567
pLKO1-shDJ-1-C	GCTGGGATTAAGGTCACCGTT	191–211	133–153
pLKO1-shDJ-1-D	ACTCTGAGAATCGTGTGGAAA	528–548	470–490

## Data Availability

Not applicable.

## Supplementary Materials

The following are available online at https://www.mdpi.com/article/10.3390/cancers13164151/s1, Table S1: The characters of colorectal cancer patients, Figure S1: The relative expression level of *DJ-1* mRNA in each tissue pair of adjacent normal tissue (N) and colorectal tumor tissue (T) from TNM stage I, II, III, IV colorectal cancer patients. *DJ-1* expression is presented as relative expression to *β-actin* for each paired colorectal cancer tissue. Figure S2: Uncropped Figure 2A. Figure S3: Uncropped Figure 4A. Figure S4: Uncropped Figure 5A. Figure S5: Uncropped Figure 5B. Figure S6: Uncropped Figure 5C.
