# Supplementary material for: Serum DJ-1 Is a Biomarker of Colorectal Cancer and DJ-1 Activates Mitophagy to Promote Colorectal Cancer Progression
Source: Cancers (Basel). 2021 Aug 18;13(16):4151. doi: 10.3390/cancers13164151 (PMC8393356; doi:10.3390/cancers13164151)
Supplement: Supplementary file 1 [file cancers-13-04151-s001.zip › cancers-1272519-supplementary.pdf]

cancers

MDPI

Supplementary Material: Serum DJ-1 is a Biomarker of Colorectal Cancer and DJ-1 Activates Mitophagy to Promote Colorectal Cancer Progression

William Tzu-Liang Chen, Han-Bin Yang, Tao-Wei Ke, Wen-Ling Liao and Shih-Ya Hung

Table S1. The characters of colorectal cancer patients.

Characters \ TNM	Stage I (*n* = 20)	Stage II (*n* = 28)	Stage III (*n* = 23)	Stage IV (*n* = 26)
**Gender**				
Male	10 (50.0%)	13 (46.4%)	16 (69.6%)	16 (61.5%)
Female	10 (50.0%)	15 (53.6%)	7 (30.4%)	10 (38.5%)
**Age (years old)**				
Mean±SE	62.2±3.2	64.1±2.0	67.6±2.7	59.6±3.0
Median (Min, Max)	61.5 (85.0, 40.0)	66.0 (47.0, 84.0)	70.0 (42.0, 87.0)	61.0 (28.0, 85.0)
**Radiotherapy**				
Yes	1 (5.0%)	3 (10.7%)	1 (4.3%)	1 (3.8%)
No	19 (95.0%)	15 (89.3%)	22 (95.7%)	25 (96.2%)
**Chemotherapy**				
Yes	1 (5.0%)	8 (28.6%)	2 (8.7%)	10 (38.5%)
No	19 (95.0%)	20 (71.4%)	21 (91.3%)	16 (61.5%)
**Targeted treatment**				
Yes	0 (0.0%)	0 (0.0%)	0 (0.0%)	1 (3.8%)
No	20 (100.0%)	28 (100.0%)	23 (100.0%)	25 (96.2%)
**Surgical management**				
Yes	20 (100.0%)	28 (100.0%)	23 (100.0%)	26 (100.0%)
No	0 (0.0%)	0 (0.0%)	0 (0.0%)	0 (0.0%)
**Lymph node invasion**				
Yes	0 (0.0%)	0 (0.0%)	23 (100.0%)	26 (100.0%)
No	20 (100.0%)	28 (100.0%)	0 (0.0%)	0 (0.0%)
**Distant metastatic**				
Yes	0 (0.0%)	0 (0.0%)	0 (100.0%)	26 (100.0%)
NO	20 (100.0%)	28 (100.0%)	23 (100.0%)	0 (0.0%)

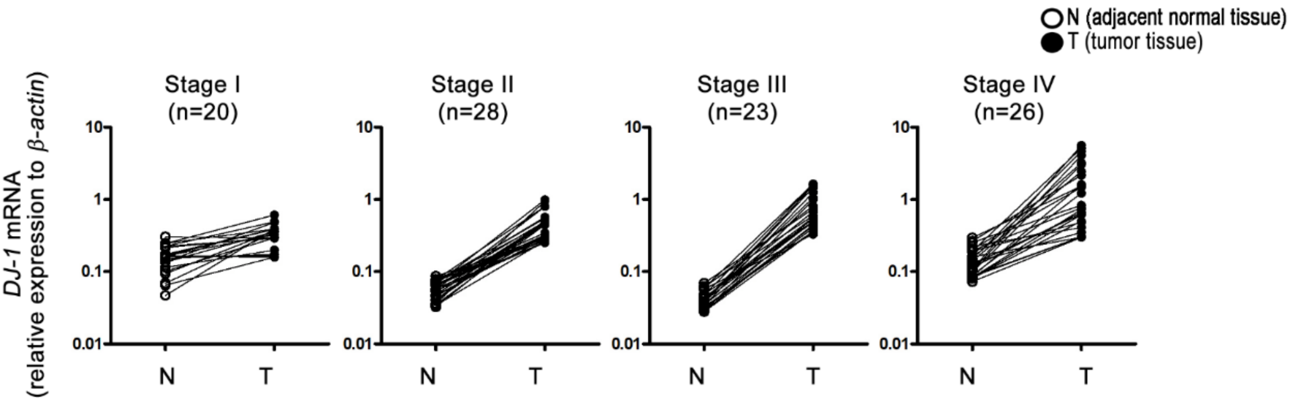

Figure S1. The relative expression level of *DJ-1* mRNA in each tissue pair of adjacent normal tissue (N) and colorectal tumor tissue (T) from TNM stage I, II, III, IV colorectal cancer patients. *DJ-1* expression in each tissue is presented as relative expression to *β-actin*.

Figure 2A

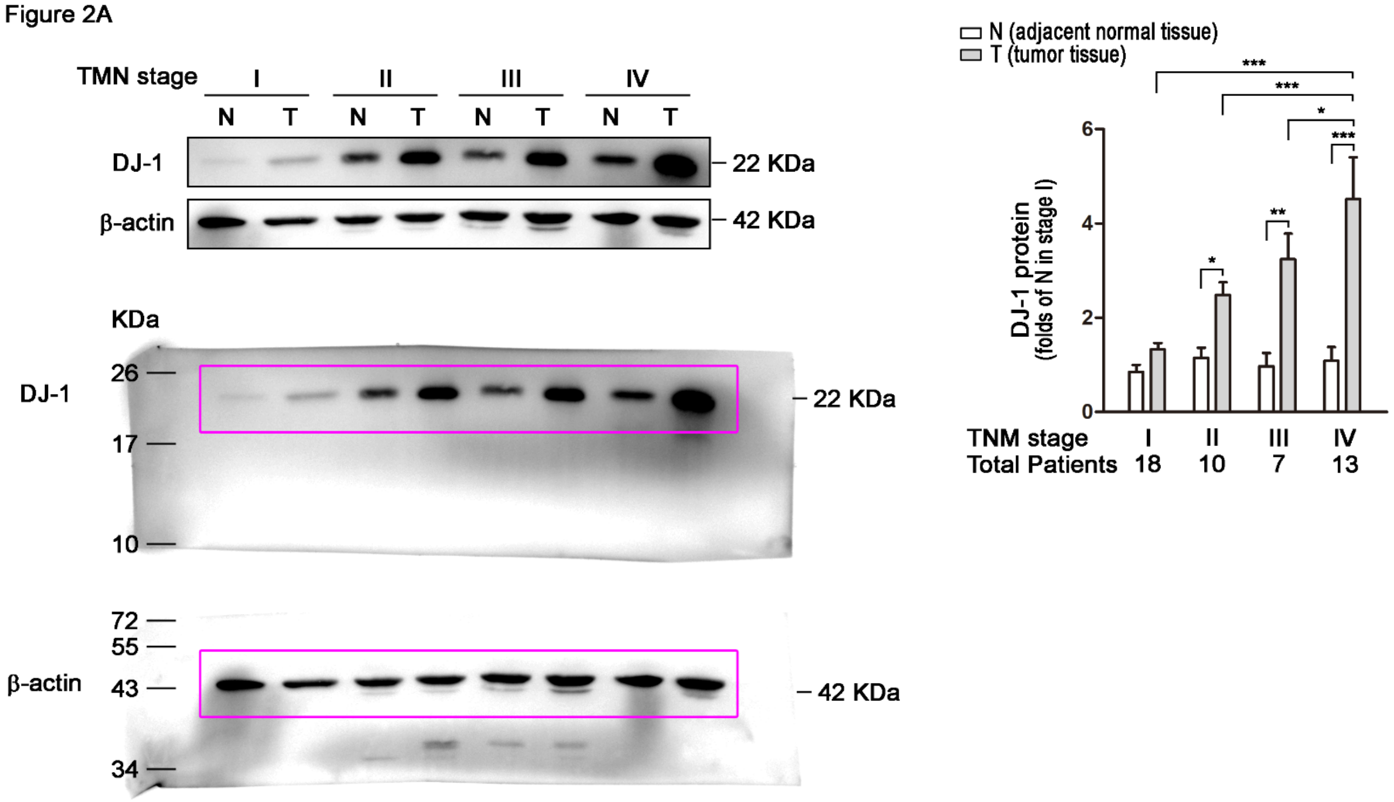

Figure S2. Uncropped Figure 2A. * The quantification data of DJ-1 protein levels are shown at the right panel. Quantitative data are expressed as the means ± S.E.M; the p-value was determined by one-way ANOVA/Newman-Keuls test. *: $p < 0.05$, **: $p < 0.01$, ***: $p < 0.001$ compared with the respectively group.

Figure 4A

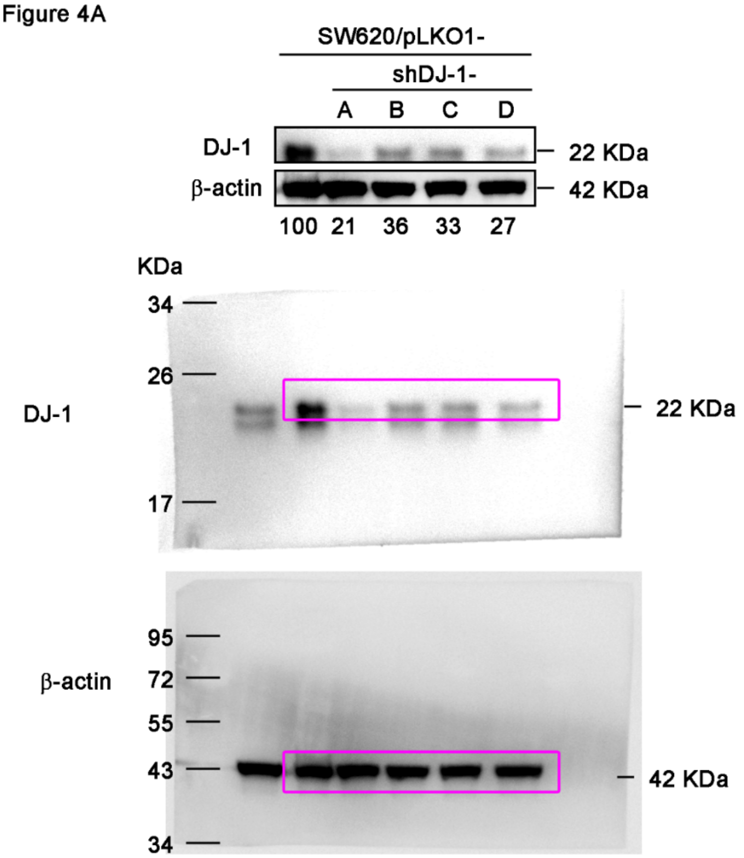

Figure S3. Uncropped Figure 4A.

Figure 5A

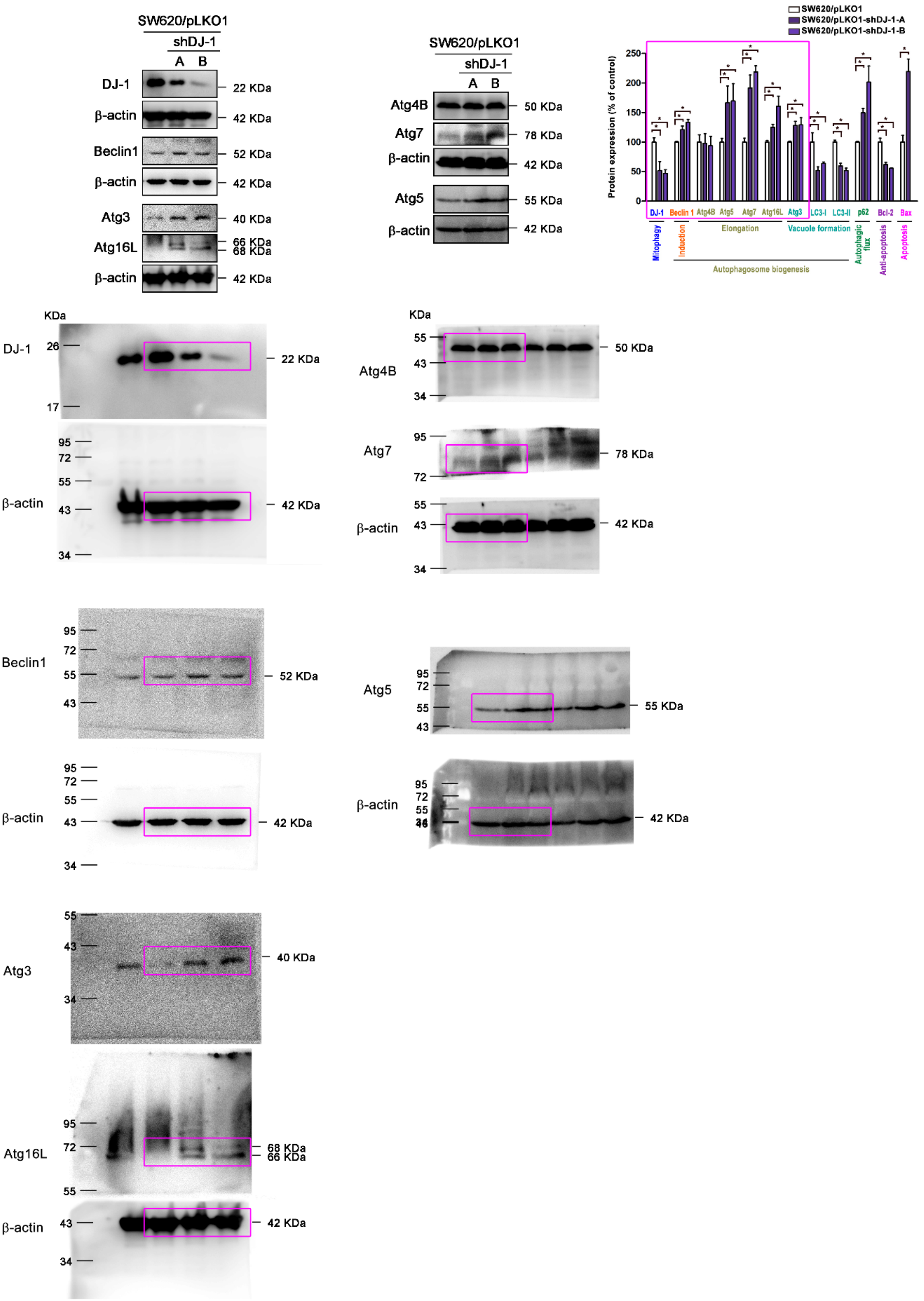

Figure S4. Uncropped Figure 5A. *The quantification data of each protein level with its function are shown here. Data are expressed as the means ± S.E.M; the Student's *t*-test determined the p-value. *: $p < 0.05$ compared with control (SW620/pLKO1).

Figure 5B

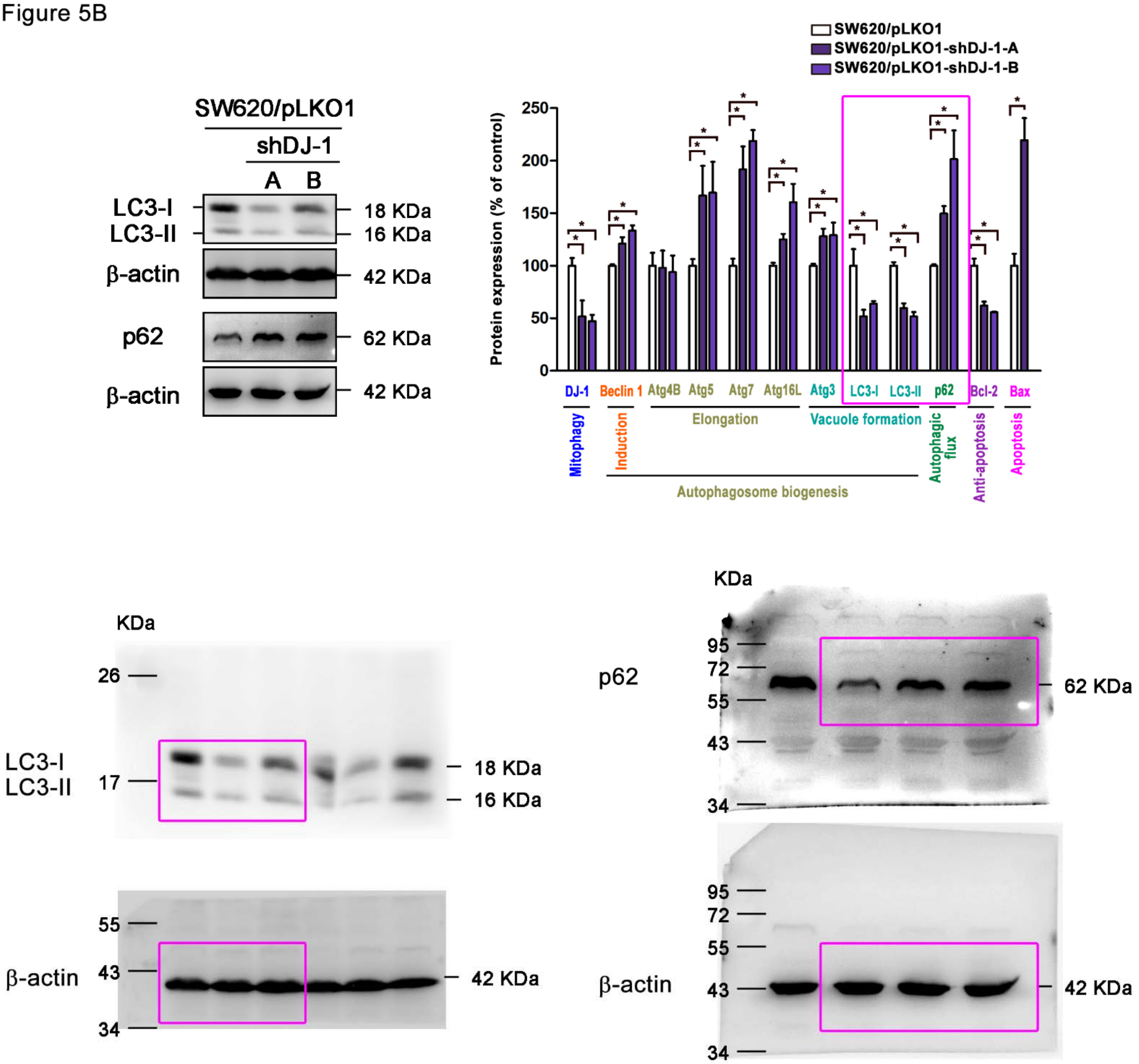

Figure S5. Uncropped Figure 5B. *The quantification data of each protein level with its function are shown here. Data are expressed as the means ± S.E.M; the Student's *t*-test determined the p-value. *: $p < 0.05$ compared with control (SW620/pLKO1).

Figure 5C

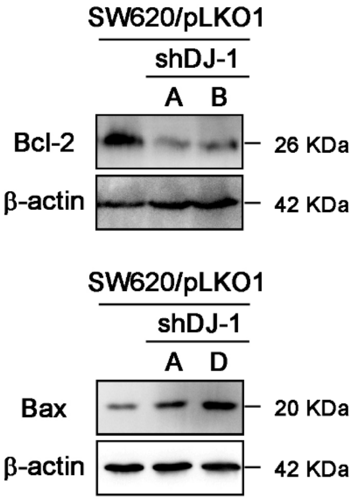

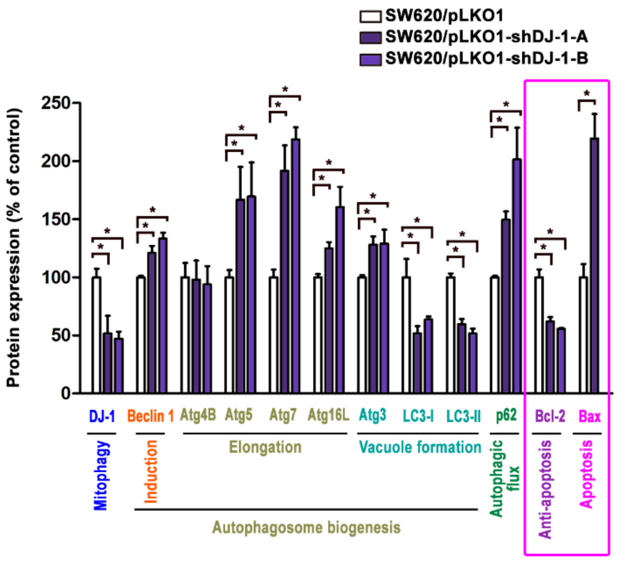

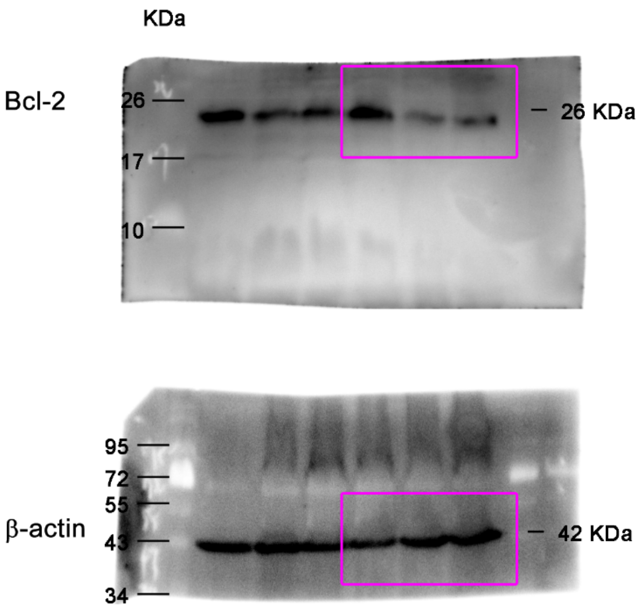

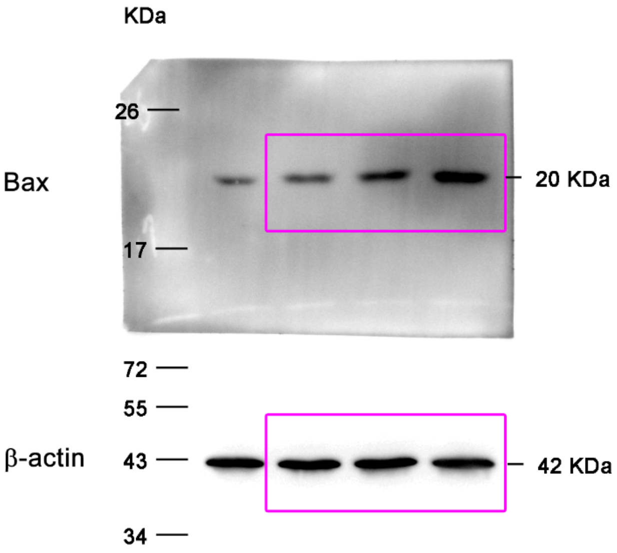

Figure S6. Uncropped Figure 5C. *The quantification data of each protein level with its function are shown here. Data are expressed as the means ± S.E.M; the Student's *t*-test determined the p-value. *: $p < 0.05$ compared with control (SW620/pLKO1).
